# Role of miR-93-5p and Its Opposing Effect of Ionizing Radiation in Non-Small Cell Lung Cancer

**DOI:** 10.1155/2024/4218464

**Published:** 2024-08-10

**Authors:** Qingtao Ni, Kai Sang, Jian Zhou, Chi Pan

**Affiliations:** ^1^ Department of Oncology Jiangsu Taizhou People's Hospital, The Affiliated Taizhou People's Hospital of Nanjing Medical University Taizhou School of Clinical Medicine Nanjing Medical University, Taizhou 225300, China; ^2^ Department of General Surgery Jiangsu Taizhou People's Hospital, The Affiliated Taizhou People's Hospital of Nanjing Medical University Taizhou School of Clinical Medicine Nanjing Medical University, Taizhou 225300, China

## Abstract

**Background:**

Radiation therapy is an effective local therapy for lung cancer. However, the interaction between genes and radiotherapy is multifaceted and intricate. Therefore, we explored the role of miR-93-5p in the proliferation, apoptosis, and migration abilities of A549 cells. Simultaneously, we also investigated the interactions between miR-93-5p and ionizing radiation (IR).

**Methods:**

Cell Counting Kit-8, transwell, and apoptotic assay were performed to measure the proliferation, migration, and apoptosis abilities. The expression levels of miR-93-5p and its target gene in lung cancer were predicted using starBase v3.0. Then, data were validated using qPCR and western blot.

**Results:**

miR-93-5p significantly promoted the proliferation (*P* < 0.01) and migration abilities (*P* < 0.001) of A549 cells. Gasdermin E (GSDME) was identified to be a putative target of miR-93-5p and had a negative correlation with miR-93-5p (*P* < 0.001). Overexpression of miR-93-5p significantly decreased GSDME in A549 (*P* < 0.001). Interestingly, miR-93-5p decreased cell proliferation (*P* < 0.01) and cell migration (*P* < 0.01) and increased apoptosis (*P* < 0.01) in A549 cells after exposure to IR.

**Conclusions:**

miR-93-5p is presumed to play an oncogenic role in lung cancer by enhancing A549 cell proliferation and migration. It can enhance the sensitivity of radiotherapy under IR conditions. We speculate that the miR-93-5p/GSDME pathway was inhibited, activating the GSDME-related pyroptosis pathway when the cells were exposed to IR. Therefore, miR-93-5p can overcome resistance to radiotherapy and improve the efficacy of radiotherapy.

## 1. Introduction

In the year 2020, an estimated 1.8 million deaths occurred worldwide from lung cancer [1]. Based on histology, lung cancer can be categorized into two major types, small-cell lung cancer (SCLC) and non-small cell lung cancer (NSCLC). Among them, NSCLC comprises ~85% of all lung cancer [2]. Lung adenocarcinoma (LUAD) and squamous cell carcinoma (LUSC) are the two main subtypes of NSCLC [3]. Although LUSC is less prevalent, its prognosis is poorer than LUAD [4]. Along with advances in medical science, targeted and immune-based therapies have provided new opportunities for patients with NSCLC. Nonetheless, radiation therapy remains an effective local therapy for lung cancer. Radiotherapy has traditionally been the standard treatment for more than half of the lung cancer patients [5].

However, the interaction between genetic factors and radiotherapy is multifaceted and intricate. Ionizing radiation (IR) leads to DNA double-strand break (DSB) in the cell genome [6]. An argument is that IR enhances the burden of small deletions, which is associated with poorer prognosis [7]. Therefore, exploring the underlying mechanisms of IR in cancers, and the interactions between genome and IR, is urgently needed to provide a potential therapeutic option for lung cancer.

MicroRNAs (miRNAs) are a class of about 22 nucleotides noncoding RNA molecules that regulate gene expression posttranscriptionally [8]. miRNAs control gene expression by binding to the 3′untranslated-region of target mRNAs [9]. Indeed, studies have revealed that almost all colorectal cancers alter miRNA expression [10]. In the context of NSCLC, miR-17 has been recognized as an epigenetic regulator of LKB1 by targeting LKB1 3′untranslated region [11]. In our previous experimental study, we identified miR-93-5p as significantly associated with breast cancer subtypes [12]. Moreover, we also found that miR-93-5p had implications for the radiosensitivity of breast cancer cells [13]. *In vivo* experiment, miR-93-5p targeted EphA4 in triple negative breast cancer (TNBC) through the NF-*κ*B pathway, and IR prevented tumor progression by inhibiting this pathway [14]. Accumulated data demonstrated that miR-93-5p enhances the malignancy of lung cancer cells [15]. However, the precise value of miR-93-5p remains unknown. Hence, this study aimed to investigate the role of miR-93-5p in the A549 cell line.

Radiation therapy is an effective treatment for late-stage NSCLC [16]. However, following radiation therapy, surviving cancer cells often convert into resistant cells, which results in local recurrence [17]. Radiation resistance also significantly impacts the survival rates of NSCLC patients [18]. Therefore, there is an urgent need to improve radiosensitivity in NSCLC. Zhang et al. [19] found that miR-29b positively affects the radiotherapy in cervical cancer. Furthermore, Maia et al. [20] found that miR-296-5p is correlated with resistance to radiotherapy in the early phase laryngeal LUSC. miR-92b enhanced the radioresistance of hepatocellular carcinoma [21]. However, no known association between miR-93-5p and IR exists in NSCLC cells. Therefore, we also explored the effects of miR-93-5p in A549 lung cancer cells exposed to IR.

## 2. Materials and Methods

### 2.1. Workflow

First, NSCLC cell line A549 was incubated with miR-93-5p mimics, miR-935p inhibitor, and negative control (NC) to detect the role of miR-93-5p. Subsequently, the expression levels of miR-93-5p and its target gene in lung cancer were predicted using starBase v3.0. Then, validation was done using qPCR and western blot. Finally, A549 cells were exposed to the appropriate dosage of IR to perform proliferation, migration, and apoptosis using CCK8, transwell, and apoptotic assay, respectively. The workflow of the study design is presented in Figure S1.

### 2.2. Cell Culture

The NSCLC cell line A549 was purchased from the American Type Culture Collection. It was cultured in Dulbecco's modified Eagle's medium (DMEM; cat. no. 10-013-CVRC, Corning, USA), 4.5 g/L glucose, L-glutamine, and sodium pyruvate, containing 10% fetal bovine serum (FBS; cat. no. SH30084.03, Hyclone). A549 cells were maintained in a 5% CO_2_ incubator at 37°C. Cells were used in a logarithmic growth phase.

### 2.3. Cell Transfection

Transfection was performed using Lipo 2000 (catalog no. 11668019; Thermo Fisher Scientific, Inc.) following the manufacturer's instructions. miR-93-5p mimics (Shenggong, Shanghai, China) and miR-93-5p inhibitor (2′OME modified; Shenggong) were cotransfected into A549 cells at a 50 nM concentration. The sequences of miR-93-5p mimics were as follows: 5′-CAAAGUGCUGUUCGUGCAGGUAG−3′ and 5′-CUACCUGCACGAACAGCACUUUG−3′. The sequences of miR-93-5 p inhibitor were as follows: 5′-CUACCUGCACGAACAGCACUUUG−3′. The transfection mix was replaced with DMEM containing 10% FBS after 4 hr.

### 2.4. Proliferation Assay

Cell proliferation assay was assessed with the Cell Counting Kit-8 (CCK8; Meilunbio, Dalian, China) assay according to the manufacturer's instructions. A549 was seeded in triplicate at a density of 10^4^ cells per well in 96-well plates. Once the cells had attached, they were transfected with miR-93-5p mimics or miR-93-5p inhibitor or NC using 50 nM of the Lipo 2000 reagent (Thermo Fisher Scientific). We conducted four sets for dose selection. First three groups were exposed to different doses of IR (2, 4, and 8 Gy), while the fourth group was used as a control. A549 cells were incubated for 48 hr after treatment. Then, 10 *µ*l of CCK8 was added to each well for 2 hr at 37°C. Subsequently, absorbance was measured at 450 nm using a Multiskan MK3 reader (Thermo Fisher Scientific Inc. MA, USA). The experiment was conducted in three replications and was repeated three times.

### 2.5. Apoptosis Assay

A549 was seeded in six-well plates at a density of 8 × 10^5^ cells per well in triplicate. After 24 hr, cells were transfected with miR-93-5p mimic, miR-93-5p inhibitor, or NC. Each treatment was irradiated 4 hr after transfection. The Annexin V-FITC/PI Apoptosis Detection Kit I (Solarbio, Science & Technology, Beijing, China) was used after 48 hr incubation with transfection liquid. The cell apoptosis was measured on a FACSalibur flow cytometer (BD Biosciences, San Jose, CA, USA).

### 2.6. Migration Assay

For cell migration analysis, the transwell assay was performed. A549 cells (4 × 10^5^ cells/ml) were seeded with serum-free DMEM into the upper chamber of an 8-*µ*m transwell. A 500 *µ*l culture medium supplemented with 20% FBS was added to the bottom chamber. Then, cells were incubated for 12 hr. The cells remaining on the polycarbonate membrane's upper surface were removed using a cotton swab. The cells in the bottom chamber were fixed with 4% paraformaldehyde for 10 min and then stained with 1% crystal violet for 5 min. The migrated cells were observed under a light microscope (magnification, ×200). The images were assessed using image analysis software (Image Pro Plus 6.0, IPP6, Media Cybernetics, MD, USA).

### 2.7. Bioinformatics Analysis

Interactions between miRNA and mRNA were predicted using starBase (http://starbase.sysu.edu.cn/index.php). Then, we performed pan-cancer analysis and survival analysis for miR-93-5p and GSDME across 32 types of cancers (10,546 miRNA-seq samples; https://rnasysu.com/encori/). First, the expression data of cancers were downloaded from the TCGA project via Genomic Data Commons Data Portal. Then, coexpression analysis for miR-93-5p and GSDME in LUSC was performed. Next, the expression values of miRNAs from miRNA-seq data and mRNA from mRNA-seq were scaled with log2 (RPM + 0.01). Finally, we analyzed GSDME as the potential miR-93-5p target gene using PITA (http://www.pictar.org/).

### 2.8. Luciferase Assay

Wild-type or mutant GSDME and miR-93-5p were amplified and cloned into the pmirGLO vector. Cells were then cotransfected with wild-type or mutant GSDME luciferase plasmid and miR-93-5p or control miRNA in 96-well plates. First, the GSDME promoter was inserted into the pGL3-basic vector. Next, miR-93-5p or miR-NC was cotransfected into pGL3-GSDME and pRL-TK vectors. Finally, luciferase activity was measured using a dual luciferase reporter system (Promega, USA).

### 2.9. Reverse Transcription (RT)-qPCR

Following transfection of A549 with miR-93-5p mimic, miR-93-5p inhibitor, or NC, total RNA was extracted using nano–magnetic beads and a MagBeads Total RNA Extraction Kit (Meixuan, Shanghai, China) as per the manufacturer's protocol. qPCR was performed in an Applied Biosystems 7500 Fast Real-Time PCR System (Applied Biosystems, Foster City, CA) under the following conditions: 10 min at 50°C, 5 min at 95°C, 15 s at 95°C, and 30 s at 60°C, for 40 cycles. The relative expression levels were calculated using the 2−*ΔΔCq* method. The internal reference gene was GAPDH. The primers used were as follows: GSDME: forward, 5′-GGTGTCAGTCCACACTCCAC-3′ and reverse, 5′-CGACCACTGGACTCGGAAAT-3′; GAPDH: forward, 5′-GCACCGTCAAGGCTGAGAAC-3′ and reverse, 5′-TGGTGAAGACGCCAGTGGA-3′. The experiment was repeated three times.

### 2.10. Irradiation

IR was generated using an X-Ray Biological Irradiator (RS2000, Rad Source Technologies). After transfection experiments, the IR groups were treated with radiation (2/4/8 Gy dose; dose rate: 1 Gy/min).

### 2.11. Western Blot Analysis

The protein expression levels of GSDME of A549 were also detected. Protein samples from A549 cells (20 *µ*g/lane) were solubilized in 12% SDS-PAGE and then transferred to polyvinylidene fluoride (PVDF) membranes. Antibodies GSDME (cat. no. 13075-1-AP; protein tech; 1 : 1000; 55 kDa) and GAPDH (cat. no. 5174; CST; 1 : 5000; 37 kDa) were diluted in 5% BSA and incubated overnight at 4°C. Next, the membranes were incubated for 2 hr with a goat antirabbit secondary antibody (cat. no. 111-035-003; Jackson ImmunoResearch Laboratories, Inc.; 1 : 5000). The membranes were finally exposed to ECL reagent (cat. no. WBKLS0500; Millipore, Bedford, MA, USA). Protein expression levels were quantified using IPP6.0 software, with GAPDH as the loading control.

### 2.12. Statistical Analysis

Data are presented as the mean ± standard deviation (SD) from three independent experiments. *P* value was calculated using SPSS version 18.0 statistical software (IBM Corp., Armonk, NY, USA). An unpaired Student's *t*-test was used to analyze the statistical difference between the two groups. One way ANOVA followed by LSD *t*-test was used to measure the statistical difference among multiple group comparisons. *P* < 0.05 was considered a statistically significant difference.

## 3. Results

### 3.1. miR-93-5p Increased the Malignant Behaviors of A549 Cells

**Figure 1:**
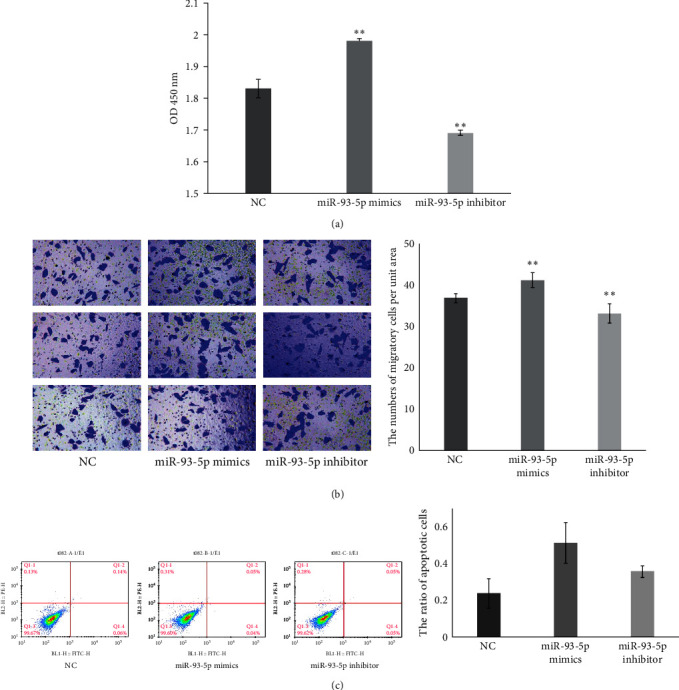
miR-93-5p increased the malignant behaviors of A549 cells. A549 cells were transiently transfected with NC, miR-93-5p mimic, or miR-93-5p inhibitor. (a) Cell proliferation after overexpression and inhibition of miR-93-5p was measured at an absorbance of 450 nm. (b) The transwell assay was conducted to determine the migratory abilities of each group in A549 cells. (c) Apoptosis was measured by annexin V-FITC/PI staining and analyzed with flow cytometry. The standard deviations from triplicate experiments are indicated in the column bar graph.  ^*∗∗*^ for *P* < 0.01. NC, negative control.

It was observed that miR-93-5p promoted the proliferation ability of A549 cells (*P*  < 0.01; Figure 1(a)). Moreover, we found miR-93-5p enhanced migration abilities (*P*  < 0.01; Figure 1(b)). Conversely, the miR-93-5p inhibitor had the opposite effect on these functional attributes (*P*  < 0.01; Figure 1). No apparent differences were observed in apoptosis assays in any group (Figure 1(c)).

### 3.2. GSDME Is a Target Gene of miR-93-5p

**Figure 2:**
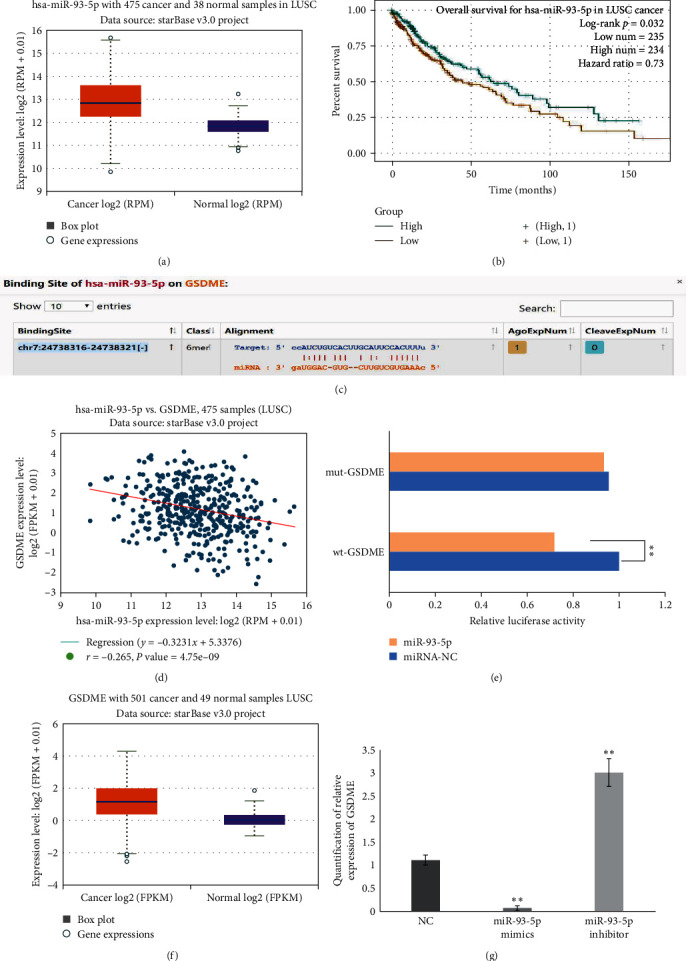
Bioinformatics results and GSDME is a target gene of miR-93-5p. miR-93-5p and its target gene were analyzed in the database. (a) The expression of miR-93-5p in LUSC samples and normal samples. (b) The prognostic value of miR-93-5p in LUSC. (c) GSDME was a putative target gene of miR-93-5p. (d) The relationship between miR-93-5p and GSDME in LUSC. (e) A double luciferase activity assay in 293T cells indicated that miR-93-5p regulates GSDME. (f) The expression of GSDME in LUSC samples and normal samples. (g) The relative expression levels of GSDME in NC, miR-93-5p mimics, and miR-93-5p inhibitors.  ^*∗∗*^ for *P* < 0.01. LUSC, lung squamous cell carcinoma; GSDME, gasdermin E.

According to bioinformatic results, high expression of miR-93-5p was found in LUSC samples compared with normal samples (*P*  < 0.001; Figure 2(a)). According to the survival analysis table of pan-cancer analysis for miR-93-5p, miR-93-5p had significant prognostic value in LUSC (Figure 2(b)). The patients with high expression of the miR-93-5p had an improved prognosis. As demonstrated in Figure 2(c), GSDME was identified to be a putative target of miR-93-5p. miR-93-5p and GSDME had a negative correlation in LUSC (*P*  < 0.001; Figure 2(d)). The results of luciferase activity assays indicated that miR-93-5p could regulate GSDME (Figure 2(e)). In addition, we found that GSDME also had high expression in LUSC samples compared with normal samples (*P*  < 0.001; Figure 2(f)).

The expression levels of GSDME in A549 cells in miR-93-5p mimic, miR-93-5p inhibitor, and NC were examined. As demonstrated in Figure 2(g), overexpression of miR-93-5p significantly downregulated the relative expression levels of GSDME compared with NC (*P*  < 0.01). At the same time, the downregulation of miR-93-5p significantly enhanced the relative expression levels of GSDME (*P*  < 0.001). These findings suggest that GSDME is likely a direct target gene of miR-93-5p.

### 3.3. miR-93-5p Enhanced the Radiosensitivity of A549 Cells

**Figure 3:**
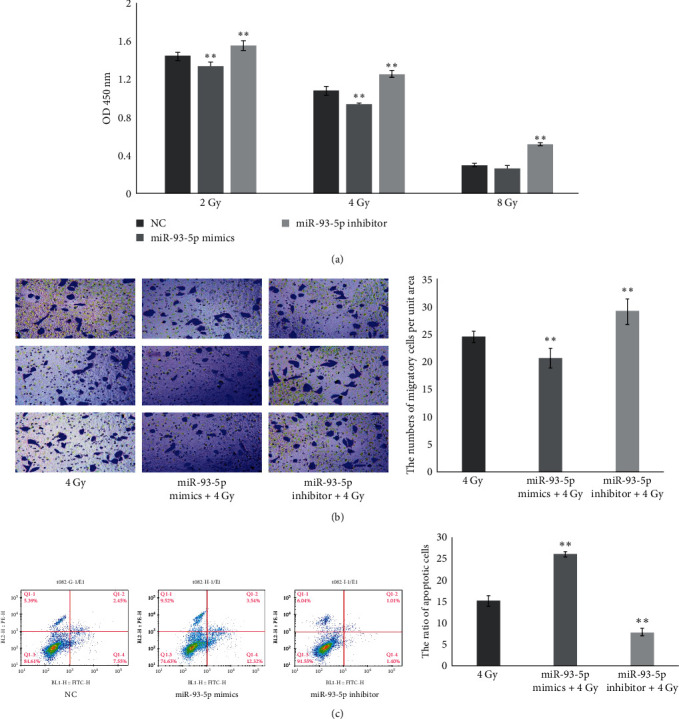
miR-93-5p enhanced the radiosensitivity of A549 cells. A549 cells were transfected by NC, miR-93-5p mimics, and miR-93-5p inhibitors. (a) Cell proliferation after overexpression and inhibition of miR-93-5p was measured at an absorbance of 450 nm with 2, 4, and 8 Gy. (b) The transwell assay was conducted to determine the migratory abilities of each group with 4 Gy in A549 cells. (c) Apoptosis was measured by annexin V-FITC/PI staining and analyzed with flow cytometry. The standard deviations from triplicate experiments are indicated in the column bar graph.  ^*∗∗*^ for *P* < 0.01. Error bars indicate the mean ± SD. NC, negative control; FITC/PI, fluorescein isothiocyanate/propidium iodide; SD, standard deviation.

We performed a cell proliferation assay to determine an appropriate dose for subsequent experiments. Three different doses of IR were explored: 2, 4, and 8 Gy. The effect of 2 Gy IR was less noticeable than that of 4 Gy. Therefore, the dose of 4 Gy was selected for subsequent experiments. These CCK8 results showed that miR-93-5p attenuated cell proliferation in A549 cells after exposure to IR with 2 and 4 Gy (*P*  < 0.01; Figure 3(a)). In addition, the number of migratory cells per unit area was markedly decreased in the miR-93-5p + 4 Gy group compared with the 4 Gy group (*P*  < 0.01; Figure 3(b)). Apoptosis assays were performed at two time points; 6 hr after transfection and 48 hr after exposure to 4 Gy IR. The miR-93-5p overexpression significantly increased the rate of apoptosis compared with the 4 Gy group (*P*  < 0.01; Figure 3(c)). In contrast, a significant decrease in the rate of apoptosis was observed in the miR-935p inhibitor compared with the 4 Gy group (*P*  < 0.01; Figure 3).

### 3.4. GSDME Was Inhibited by miR-93-5p but Increased by IR

**Figure 4:**
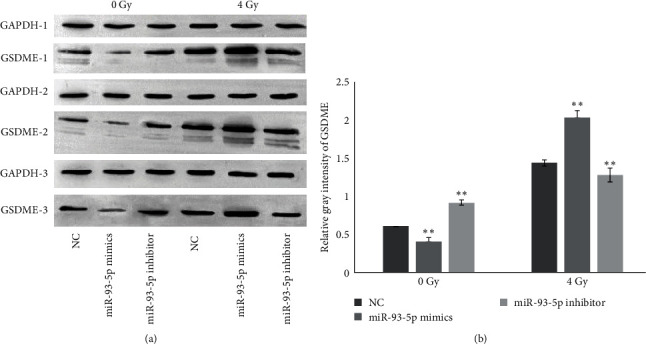
GSDME was inhibited by miR-93-5p but increased by IR. (a) Representative western blot of GSDME in NC, miR-93-5p mimics, and miR-93-5p inhibitors with or without IR in A549 cells. (b) The protein expression levels of GSDME in NC, miR-93-5p mimics, and miR-93-5p inhibitors with or without IR in A549 cells. The standard deviations from triplicate experiments are indicated in the column bar graph.  ^*∗∗*^ for *P* < 0.01. Error bars indicate the mean ± SD. GSDME, gasdermin E; NC, negative control; IR, ionizing radiation; SD, standard deviation.

The overexpression of miR-93-5p significantly downregulated the protein expression levels of GSDME compared with NC (*P* < 0.01; Figure 4). This finding was consistent with the mRNA expression levels of GSDME among these groups in Figure 2(f). Following 4 Gy IR exposure to the A549 cell line, the gray values of GSDME in the miR-93-5p group were significantly higher than in the 4 Gy group (*P*  < 0.01; Figure 4). In contrast, there was a significant decrease in the miR-93-5p inhibitor group compared with the 4 Gy group (*P*  < 0.01; Figure 4).

## 4. Discussion

miRNA participates in biological functions in various cancers by regulating protein-coding genes [22]. For example, miR-93-5p reverses the resistance of SCLC to chemotherapy [23]. In the present study, we demonstrated the value of miR-93-5p in A549 cells. We found that overexpression of miR-93-5p enhanced the migratory ability and proliferation. In contrast, the downregulation of miR-93-5p had the opposite role. These observations are consistent with the findings of Yang et al. [24] who also identified miR-93-5p as an oncogenic factor. These findings provide strong evidence that miR-93-5p is involved in mediating cellular functions.

In the present study, we have demonstrated the significance of miR-93-5p negatively correlated with GSDME in LUSC. Moreover, GSDME was a target gene of miR-93-5p. GSDME is a member of the gasdermin family, a gene originally implicated in hereditary hearing loss. A link between GSDME and cancer death has been established [25]. Moreover, GSDME can switch caspase-3-mediated apoptosis to pyroptosis [26]. Therefore, we explored the correlations between miR-93-5p and prognosis in LUSC. High expression of miR-93-5p was observed, and it had significant prognostic value in LUSC. According to the literature, miR-93-5p is highly expressed in NSCLC [24, 27], consistent with our result. Consequently, we hypothesize that miR-93-5p induces cell proliferation and migration by targeting the GSDME.

We discovered that after IR exposure, miR-93-5p strongly influenced cell apoptosis in A549 cells compared to IR exposure alone. Moreover, the results also demonstrated that miR-93-5p decreased cell proliferation and migration and increased apoptosis in A549 cells after exposure to IR. This result is the evidence that points toward miR-93-5p enhancing the sensitivity of radiotherapy in A549 cells. It indicates that IR induces the exact opposite role of miR-93-5p. The mechanism of this identity shift is still unknown.

The most important limitation of our study is insufficient evidence. The mechanism only allows for speculation. Thus, we can only conjecture about the unique priming mechanism of IR. IR often results in DNA DSB in genome [6], small deletions [7], and cell death. As a result, we assumed that when cells were exposed to IR, the miR-93-5p/GSDME pathway was inhibited, resulting in GSDME-related pyroptosis pathway activation. The interaction between GSDME and miR-93-5p may result in the depletion of miR-93-5p, ultimately diminishing its effect. Moreover, we found that the high expression of miR-93-5p was correlated with a favorable prognosis in LUSC, with most LUSC patients receiving radiotherapy. The results of bioinformatics analysis provided further confirmation for our hypothesis. However, the specific mechanism of IR and miR-93-5p/GSDME is unclear and still needs further research and experiment verification.

From the standpoint of miR-93-5p function, these findings revealed that radiosensitivity increased as miR-93-5p levels increased in A549 cells. Correspondingly, miR-93-5p inhibitor enables the cells to be more tolerant to radiotherapy. Our previous experiments are consistent with these findings in breast cancer cells [13]. In short, miR-93-5p is a potential target gene in NSCLC. However, little is known regarding the underlying mechanism of miR-93-5p in the radiotherapy of NSCLC patients. Notably, pyroptosis has not yet to be studied in terms of cancer radiosensitivity.

In summary, miR-93-5p plays an oncogenic role in lung cancer by enhancing A549 cell proliferation and migration. Furthermore, it can enhance the sensitivity of radiotherapy under IR conditions. We speculate that the miR-93-5p/GSDME pathway was inhibited, resulting in GSDME-related pyroptosis pathway activation when the cells were exposed to IR. miR-93-5p helps overcome resistance to radiotherapy and improves the efficacy of radiotherapy. It can be combined with radiotherapy to develop new treatment options for NSCLC.

## Data Availability

The data that support the findings of this study are available from the corresponding author upon reasonable request.

## Abbreviations

CCK8: Cell Counting Kit-8
DMEM: Dulbecco's modified Eagle's medium
DSB: Double-strand break
FBS: Fetal bovine serum
GSDME: Gasdermin E
IR: Ionizing radiation
LUAD: Lung adenocarcinoma
LUSC: Lung squamous cell carcinoma
miRNAs: microRNAs
NC: Negative control
NSCLC: Non-small cell lung cancer
PVDF: Polyvinylidenfluoride
RT: Reverse transcription
TNBC: Triple negative breast cancer
SCLC: Small-cell lung cancer
SD: Standard deviation.

## Supplementary Materials

Supplementary Materials Figure S1: Workflow of the present study.
