# Supplementary figures and images for: Role of miR-93-5p and Its Opposing Effect of Ionizing Radiation in Non-Small Cell Lung Cancer
Source: Anal Cell Pathol (Amst). 2024 Aug 10;2024:4218464. doi: 10.1155/2024/4218464 (PMC11330335; doi:10.1155/2024/4218464)

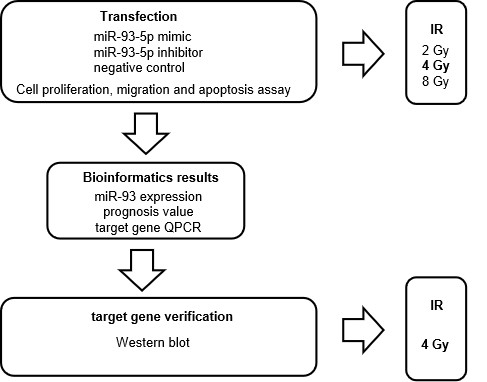

Supplement: Supplementary Materials — Figure S1: Workflow of the present study. [file 4218464.f1.jpg]
